# Alternative cleavage and polyadenylation in spermatogenesis connects chromatin regulation with post-transcriptional control

**DOI:** 10.1186/s12915-016-0229-6

**Published:** 2016-01-22

**Authors:** Wencheng Li, Ji Yeon Park, Dinghai Zheng, Mainul Hoque, Ghassan Yehia, Bin Tian

**Affiliations:** Department of Microbiology, Biochemistry and Molecular Genetics, Rutgers New Jersey Medical School, Newark, NJ USA; Transgenic Core Facility, Rutgers New Jersey Medical School, Newark, NJ USA; Rutgers Cancer Institute of New Jersey, Newark, NJ USA

**Keywords:** Alternative polyadenylation, Testis, 3’UTR, Chromatin, Transcription, Post-transcriptional control, Transposable elements

## Abstract

**Background:**

Most mammalian genes display alternative cleavage and polyadenylation (APA). Previous studies have indicated preferential expression of APA isoforms with short 3’ untranslated regions (3’UTRs) in testes.

**Results:**

By deep sequencing of the 3’ end region of poly(A) + transcripts, we report widespread shortening of 3’UTR through APA during the first wave of spermatogenesis in mouse, with 3’UTR size being the shortest in spermatids. Using genes without APA as a control, we show that shortening of 3’UTR eliminates destabilizing elements, such as U-rich elements and transposable elements, which appear highly potent during spermatogenesis. We additionally found widespread regulation of APA events in introns and exons that can affect the coding sequence of transcripts and global activation of antisense transcripts upstream of the transcription start site, suggesting modulation of splicing and initiation of transcription during spermatogenesis. Importantly, genes that display significant 3’UTR shortening tend to have functions critical for further sperm maturation, and testis-specific genes display greater 3’UTR shortening than ubiquitously expressed ones, indicating functional relevance of APA to spermatogenesis. Interestingly, genes with shortened 3’UTRs tend to have higher RNA polymerase II and H3K4me3 levels in spermatids as compared to spermatocytes, features previously known to be associated with open chromatin state.

**Conclusions:**

Our data suggest that open chromatin may create a favorable cis environment for 3’ end processing, leading to global shortening of 3’UTR during spermatogenesis. mRNAs with shortened 3’UTRs are relatively stable thanks to evasion of powerful mRNA degradation mechanisms acting on 3’UTR elements. Stable mRNAs generated in spermatids may be important for protein production at later stages of sperm maturation, when transcription is globally halted.

**Electronic supplementary material:**

The online version of this article (doi:10.1186/s12915-016-0229-6) contains supplementary material, which is available to authorized users.

## Background

Pre-mRNA cleavage/polyadenylation (C/P) is a 3’ end processing mechanism employed in eukaryotes for expression of almost all protein-coding transcripts and long non-coding RNAs by RNA polymerase II (RNAPII) [1, 2]. The site for C/P, commonly known as the polyA site or pA, is defined by both upstream and downstream cis elements [3, 4]. In mammals, the cis elements include the polyadenylation signal (PAS), such as AAUAAA, AUUAAA, or close variants, located within ~40 nucleotides (nt) from the pA; UGUA elements, typically located upstream of the PAS; U-rich elements located around the PAS; and downstream U-rich and GU-rich elements, generally located within ~100 nt from the pA. The C/P machinery in mammalian cells is composed of over 20 core factors [5–7]. Some form subcomplexes, including cleavage and polyadenylation specificity factor (CPSF), containing CPSF-160, CPSF-100, CPSF-73, CPSF-30, Fip1 and WDR33; cleavage stimulation factor (CstF), containing CstF-77, CstF-64/CstF-64τ and CstF-50; cleavage factor I (CFI), containing CFI-68 or CFI-59 and CFI-25; and cleavage factor II (CFII), containing Pcf11 and Clp1. Single proteins involved in C/P include symplekin, poly(A) polymerases (PAPs), nuclear poly(A) binding protein (PABPN1) and RNAPII. In addition, RBBP6, PP1α and PP1β are present in the C/P complex and homologous to yeast C/P factors [6, 8].

Most mammalian genes express alternative cleavage and polyadenylation (APA) isoforms [9–12]. APA in the 3’ untranslated region (3’UTR), called 3’UTR-APA, leads to isoforms with different 3’UTR lengths. Because the 3’UTR plays important roles in mRNA metabolism, 3’UTR-APA can impact the post-transcriptional control of gene expression [13–15]. Studies have shown that the APA pattern of genes is tissue-specific [10, 16–18]. For example, long and short 3’UTR APA isoforms tend to be expressed in the brain and testis, respectively [10, 16, 19, 20]. In addition, APA is regulated in cell proliferation, differentiation and development [11, 20–22], as well as in response to extracellular signals [23]. A growing number of mechanisms have been found to modulate APA, including core C/P factor activities, binding of RNA-binding proteins (RBPs) near the pA, splicing and transcriptional controls, etc. [13, 14, 24].

A sizable fraction of mammalian genes display APA in introns and exons, which affects coding sequences (CDS) [12, 25]. These APA events are called CDS-APA and are largely controlled by the dynamic competition between splicing and C/P [25, 26]. However, transcripts utilizing pAs located near promoters are specifically controlled by U1 snRNP [27], which has been implicated in controlling transcriptional directionality [28]. On the other hand, a large fraction of promoters lead to transcription of short antisense transcripts using pAs near the transcription start site (TSS). These transcripts are commonly known as promoter-upstream transcripts (PROMPTs) or upstream antisense RNAs (uaRNAs), and are subject to the regulation by the exosome/PABPN1 [26, 29].

**Fig. 1:**
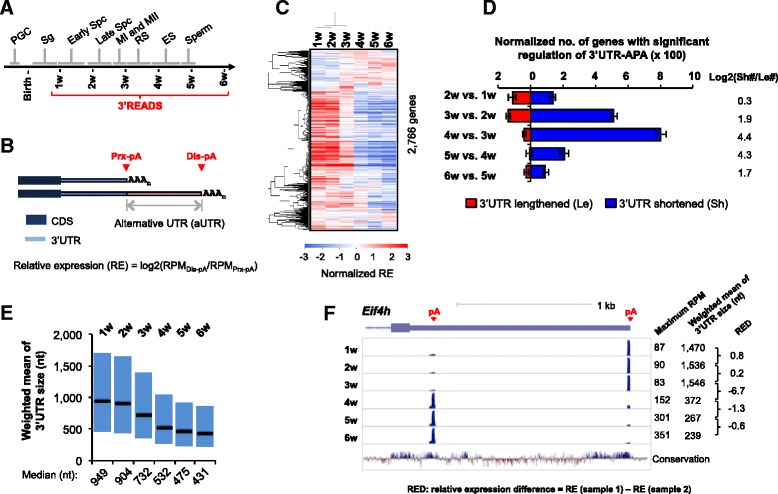
Significant 3’UTR shortening during spermatogenesis. **a** Schematic of the first wave spermatogenesis in mouse. ES, elongated spermatids; MI and MII, meiosis phases I and II; PGC, primordial germ cells; RS, round spermatids; Sg, spermatogonia; Spc, spermatocytes. **b** Schematic of APA analysis. Relative expression (RE) of two selected pA isoforms using proximal (Prx) and distal (Dis) pAs was based on the formula indicated. The region between the two pAs is alternative UTR (aUTR). **c** Heatmap showing gene RE values during spermatogenesis. Each row is a pA pair of a gene, whose RE values in different samples (columns) were normalized to the mean of each row. Two-way clustering was conducted using the Pearson correlation coefficient as metric. Only genes with read number ≥20 for Prx-pA and Dis-pA isoforms combined were selected for analysis (a total of 2,766 genes). **d** Normalized number of genes with significant regulation of 3’UTR-APA between adjacent stages. The global analysis of alternative polyadenylation (GAAP) and significance analysis of alternative polyadenylation (SAAP) methods were used (see Methods for details). The numbers of genes with 3’UTR lengthened (Le) and shortened (Sh) are plotted separately, as indicated. Error bars are standard deviation based on 20 times of data sampling. The log2Ratio of number of Sh genes to that of Le genes, or log2(Sh#/Le#), for each comparison is shown on the right. **e** Boxplots of weighted mean of 3’UTR size (see Methods for its calculation). Median values are indicated. **f** APA of an example gene *Eif4h* in spermatogenesis. Only the 3’-most exon is shown. The maximum RPM value for each track (y-axis) and the weighted 3’UTR size at each time point are shown. The relative expression difference (RED) value between two adjacent time points is indicated. The conservation track was based on the PhyloP score

Spermatogenesis is the developmental process to generate mature sperms from spermatogonia (illustrated in Fig. 1a), the undifferentiated male germ cells. Spermatogenesis in mouse takes about 30 days, and begins with a mitotic phase, during which spermatogonia become spermatocytes. This is followed by a meiotic phase, during which diploid spermatocytes become haploid round spermatids. Round spermatids then undergo substantial cell transformation and morphology changes, a process called spermiogenesis, during which cells first become elongating spermatids and then mature spermatozoa with acrosome and sperm tail. Because the first wave spermatogenesis after birth is largely synchronized, longitudinal examination of the testis in the first several weeks effectively reveals cells at different stages of spermatogenesis. For example, germ cells in the testis of a newborn mouse are arrested at the G0 and G1 stages of first mitosis, and differentiate into spermatogonia by 6 days post partum (dpp). Some spermatogonia differentiate into spermatocytes around ~10 dpp. The first meiotic division (MI) starts ~12 dpp and completes by 21 dpp, which is followed by a rapid second cell division (MII) and then spermiogenesis.

Spermatogenesis involves substantial regulation of gene expression at multiple levels. First, there is pervasive transcription in spermatocytes and spermatids [30, 31], which is attributable to permissive open chromatin state, as indicated by histone marks such as H3K4 methylation [30, 32]. Nuclear DNA condenses during differentiation into elongating spermatids, leading to halting of transcriptional activity. As such, protein expression in the later phase of spermiogenesis relies on the mRNAs transcribed earlier [33]. In addition, RNA processing is highly regulated during spermatogenesis, as indicated by rampant alternative splicing changes [31, 34–37] and alternative polyadenylation changes [16, 38–40].

One of the major players in gene expression regulation during spermatogenesis is Piwi-interacting small RNA (piRNA) [41]. Two waves of piRNAs are expressed during the process, i.e., pre-pachytene piRNAs and pachytene piRNAs. While the former has been implicated in inhibition of transposable elements (TEs) through epigenetic mechanisms [42, 43], the latter has recently been shown to cause degradation of mRNA through the function of Miwi [44–47]. Inhibition of TE expression is important for germ cells, because uncontrolled expression can lead to genome instability [48].

We and others have previously found that pAs usage in testis is rather unique as compared to other tissues [10, 16, 39]. In general, transcripts with short 3’UTRs tend to be expressed in testis. However, how this APA pattern fits into the timeline of spermatogenesis is unclear, nor are the mechanisms and consequences. In addition, how CDS-APA events, which account for over 40 % of all APA events in the mouse genome [12], are regulated in spermatogenesis has never been studied. Further, we recently found that many bidirectional promoters express uaRNAs with pAs located within 2 kb from the promoter [26]. How these uaRNAs are regulated in spermatogenesis is completely unknown. Here we examine APA isoform expression in the first wave of spermatogenesis to identify the cell type that has the shortest 3’UTRs and reveal potential mechanisms behind the global 3’UTR shortening. We also analyze CDS-APA and uaRNA expression. We present evidence that 3’UTR-APA regulation is important for RNA metabolism in sperm maturation.

## Results

### Significant 3’UTR shortening in spermatogenesis

To examine how APA is regulated during spermatogenesis, we extracted RNA from mouse testes at different time points after birth, namely 1 week (w), 2w, 3w, 4w, 5w and 6w. These time points correspond to key phases in the first wave of spermatogenesis (indicated in Fig. 1a), during which cells are largely synchronized [49, 50]. RNA was subjected to 3’ region extraction and deep sequencing (3’READS), a deep sequencing method we recently developed to examine the expression of poly(A) + RNAs and identify their pAs [12].

We first compared the relative expression of the top two most abundant 3’UTR-APA isoforms of each gene at different time points. Based on the location of their pAs relative to the coding region, they were named proximal pA (Prx-pA) isoform and distal pA (Dis-pA) isoform, respectively (illustrated in Fig. 1b). Using relative expression (RE) values between Prx-pA and Dis-pA isoforms (Fig. 1b), we found that the relative expression of Dis-pA isoforms to Prx-pA isoforms were significantly lower in 4–6 week samples than in 1–3 week samples (Fig. 1c). Consistently, 1–3 week samples could be separated from 4–6 week samples using cluster analysis based on RE values (Fig. 1c).

We next applied significant analysis of alternative polyadenylation (SAAP), our recently developed method to statistically identify regulated APA events using a bootstrapping approach [26], and global analysis of alternative polyadenylation (GAAP), a method for comparing the extent of APA regulation between samples using a read sampling method [26]. With sequencing depth controlled across all samples (1.5 M) and false discovery rate (FDR) set at 5 %, we found that 3’UTRs began to shorten between 3w and 2w, and significant shortening took place between 4w and 3w as well as between 5w and 4w, as indicated by the ratio of number of genes with shortened 3’UTRs (Sh) to that with lengthened 3’UTRs (Le) (Fig. 1d, right). While 4w vs. 3w and 5w vs. 4w were similar in the extent of regulation (log2(Sh#/Le#) = 4.4 and 4.3, respectively), the former involved much more APA events than the latter (by ~4-fold, Fig. 1d, left). To examine 3’UTR length changes more directly, we calculated the 3’UTR size for each gene in each sample using weighted mean of all 3’UTR-APA isoforms, with weight being the read number in the sample. In line with other analysis results, the median 3’UTR size decreased progressively from 949 nt at 1w to 431 nt at 6w (Fig. 1e). An example gene *Eif4h* is shown in Fig. 1f, which had two detected pA isoforms and displayed substantial 3’UTR shortening in spermatogenesis (weighted mean of 3’UTR size = 1,470 nt and 239 nt at 1w and 6w, respectively). Consistent with the global trend, the period between 3w and 4w involved a switch-like change of isoform expression (Fig. 1f). This is also supported by the relative expression difference (RED) value, which is the difference between RE values from two adjacent time points (Fig. 1f).

**Fig. 2:**
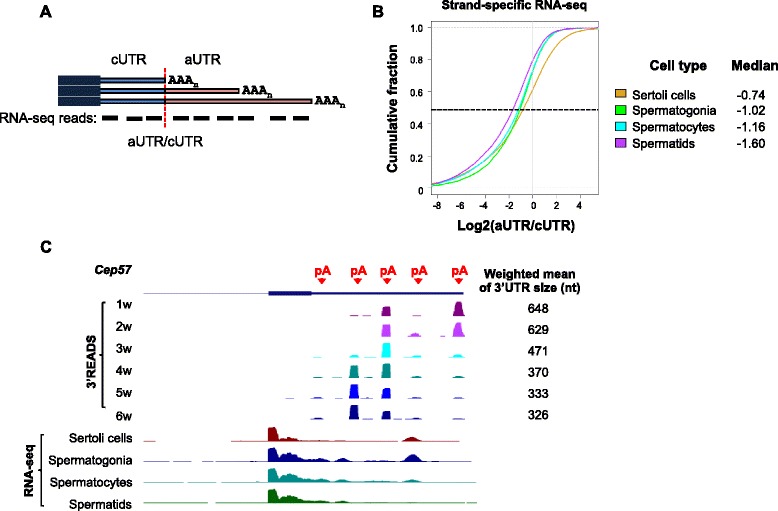
3’UTRs are the shortest in spermatids. **a** Schematic showing analysis of 3’UTR-APA using RNA-seq reads. Three 3’UTR-APA isoforms are shown. The common 3’UTR portion is called cUTR, and the alternative portion aUTR. RNA-seq reads mapped to aUTR was normalized to those mapped to cUTR to infer the relative expression of long vs. short 3’UTR isoforms. **b** Cumulative distribution function (CDF) curves of log2Ratio for RNA-seq reads mapped to aUTRs vs. those to cUTRs in different purified cells from testis. Median values are shown on the right. The data set used [NCBI GEO:GSE43717] was based on a strand-specific RNA-seq method. **c** An example gene *Cep57*. The 3’READS data indicating 3’UTR-pA sites are shown on the top and RNA-seq data are shown at the bottom. Weighted mean of 3’UTR size based on the 3’READS data is shown for each time point

There are several cell types in the testis, including spermatogonia, spermatocytes, spermatids, mature sperms and Sertoli cells. Since 2w testes are highly enriched with spermatocytes and 4w testes with spermatids, our result suggests significant 3’UTR shortening during maturation of spermatocytes into spermatids. To confirm cell specificity of APA, we analyzed a strand-specific RNA-seq data set with different cell types purified from the testis [30]. Because RNA-seq data do not directly reveal APA isoforms, we used our 3’READS data to divide 3’UTRs into two portions (Fig. 2a): the region before the first pA, named common UTR (cUTR), and the rest of 3’UTR, named alternative UTR (aUTR). We then calculated the ratio of RNA-seq read density in aUTR to that in cUTR, shown as log2(aUTR/cUTR), to reflect the expression level of long 3’UTR isoforms relative to the short 3’UTR isoforms (Fig. 2a). Consistent with the 3’READS data, the median log2(aUTR/cUTR) ratio progressively decreased from spermatogonia to spermatocytes to spermatids (Fig. 2b). The value for Sertoli cells was the highest, indicating much longer 3’UTRs in these cells. An example gene *Cep57* is shown in Fig. 2c, which displayed fewer RNA-seq reads mapped to the downstream region of the first 3’UTR pA in spermatids compared to other cell types (Fig. 2c, bottom). Note that the 3’READS data provided a much more precise picture of pAs and their regulation (Fig. 2c, top), attesting to the superiority of the method over a regular RNA-seq method for APA analysis. Taken together with the 3’READS result, these data indicate that 3’UTRs progressively shorten during spermatogenesis, with the shortest 3’UTRs being expressed in spermatids.

### 3’UTR shortening is coupled with upregulation of gene expression that is important for sperm maturation

**Fig. 3:**
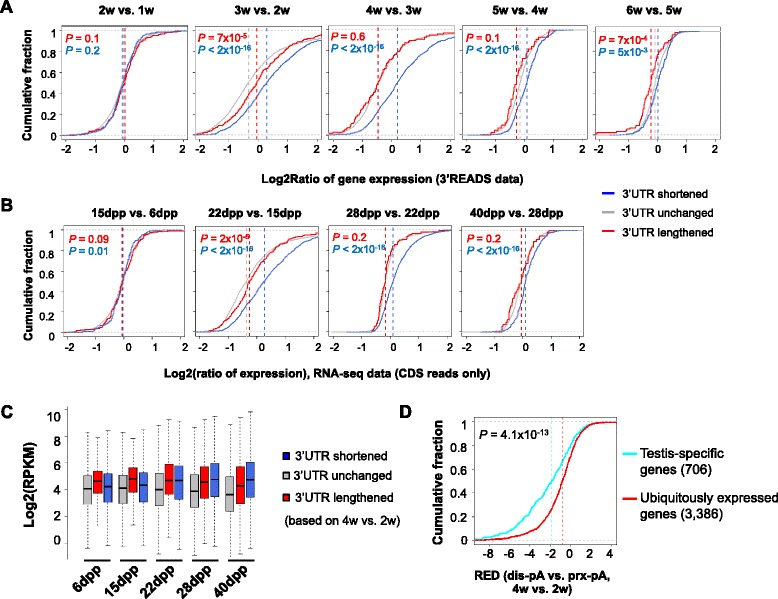
Genes with shortened 3’UTRs are more likely to have upregulated expression. **a** Gene expression changes at different stages of spermatogenesis. Genes were divided into three groups based on 3’UTR regulation between comparing samples (FDR = 5 %, SAAP analysis), namely shortened, unchanged and lengthened (shown in blue, gray and red, respectively). 3’READS data were used for the analysis, with all APA isoforms of a gene combined to represent the overall expression of the gene. The median value for each group is indicated by a dotted vertical line. *P* values (K–S test) indicating difference in expression between genes with shortened or lengthened 3’UTRs and genes with 3’UTRs unchanged are shown in each graph (in blue or red, respectively). **b** Gene expression changes between different stages of spermatogenesis based on RNA-seq reads [NCBI GEO:GSE42004] mapped to coding sequences (CDS). Because only CDS reads were used, gene expression analysis was not affected by 3’UTR changes. As in (**a**), genes were divided into three groups based on APA regulation using 3’READS data with the closest time points. Dpp, day post partum. **c** Gene expression levels (log2(RPKM)) for genes with shortened, unchanged or lengthened 3’UTRs at different stages of spermatogenesis. The plot is based on the RNA-seq data used for (**b**). 3’UTR regulation is based on 4w vs. 2w comparison. **d** Testis-specific genes tend to have greater 3’UTR shortening than ubiquitously expressed genes. The number of genes for each group is indicated. The *P* value (K–S test) indicates difference in relative expression difference (RED, 4w vs. 2w, see Fig. 1f for definition) between two groups

We next asked whether 3’UTR shortening in spermatogenesis is related to gene expression changes. To this end, we first summed up all 3’UTR-APA isoforms to represent gene expression and examined changes between adjacent time points. Compared to genes without 3’UTR changes, genes with shortened 3’UTRs were significantly upregulated in spermatogenesis (blue vs. gray lines in Fig. 3a). This difference could be observed between any adjacent time points after 2 weeks, with 3w vs. 2w and 4w vs. 3w being the most significant ones (*P* <2 × 10^−16^, Kolmogorov–Smirnov, or K–S, test). To corroborate this result, we analyzed a RNA-seq data set, for which RNAs from testes were sequenced by a strand-specific method [44]. We used only reads mapped to CDS to eliminate the possible influence of 3’UTR change on gene expression calculation (Fig. 3b). Consistent with the 3’READS result, genes with shortened 3’UTRs were significantly upregulated from 15 dpp to 40 dpp, as compared to genes without 3’UTR regulation or with lengthened 3’UTRs (Fig. 3b).

Interestingly, we found that the absolute expression level, as indicated by CDS read density (log2(RPKM)), of the genes with 3’UTR shortening was significantly higher than that of genes without 3’UTR changes, particularly at later stages of spermatogenesis (*P* = 0 for 28 dpp and 40 dpp samples, K–S test, Fig. 3c), suggesting that genes with shortened 3’UTRs are important for functions in spermatogenesis. Consistently, the top gene ontology (GO) terms associated with genes with 3’UTR shortening were highly relevant to sperm maturation, such as “protein ubiquitination”, “calcium ion import”, “centrosome”, “ciliary part”, etc. (Table 1). Notably, the protein ubiquitination pathway, which has been implicated in playing key roles in spermiogenesis, such as nucleosome removal and morphogenesis of the sperm [51], was also found to be highly significant by the ingenuity pathway analysis (Additional file 1: Table S1). In further support of the functional relevance of APA to sperm development, we found that testis-specific genes tend to have significantly greater 3’UTR shortening (4w vs. 2w) than genes ubiquitously expressed across different tissues (*P* = 4.1 × 10^−13^, K–S test, Fig. 3d). Taken together, these data indicate that 3’UTR shortening coordinates with upregulation of genes that play important roles in sperm maturation.

**Table 1:** Gene ontology terms enriched for genes with significant 3’UTR shortening in spermatogenesis

−log_10_(*P*)	Gene ontology terms
Biological process	
3.5	protein ubiquitination
3.2	calcium ion import
2.4	ethanolamine-containing compound metabolic process
2.3	protein K48-linked deubiquitination
2.3	cilium organization
Cellular component	
4.0	centrosome
3.8	ciliary part
2.8	cell junction
2.8	microtubule associated complex
2.6	lipid particle

APA regulation was based on comparison of 4w vs. 2w samples. Only top five terms for each of the two gene ontology (GO) categories are shown. *P* is based on the Fisher’s exact test

### 3’UTR shortening correlates with high RNA polymerase II signals and open chromatin state

**Fig. 4:**
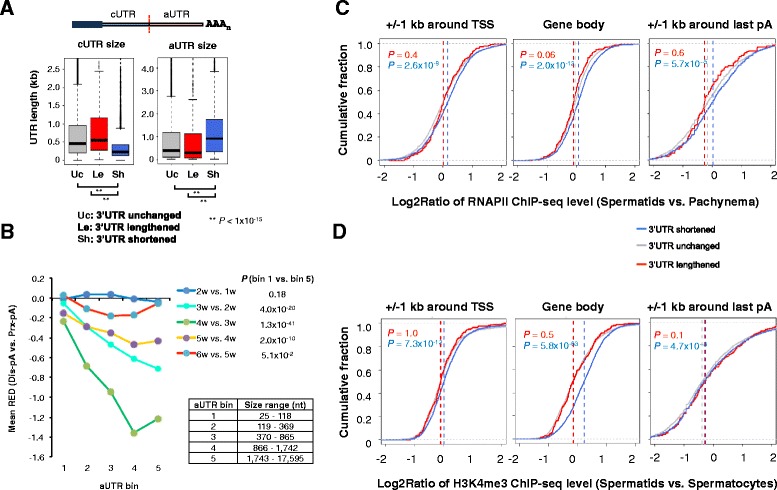
Shortening of 3’UTR by APA correlates with high transcriptional activity and open chromatin. **a** cUTR and aUTR sizes of genes with different 3’UTR-APA regulations. Left, boxplot showing cUTR size of genes with shortened (Sh), lengthened (Le), or unchanged (Uc) 3’UTRs. Right, same as the plot on the left except that aUTR size is plotted. 3’UTR regulation by APA was based on the SAAP analysis (4w vs. 2w, FDR = 5 %). *P* values comparing different gene groups were based on the K–S test. **b** Genes with longer aUTRs tend to have greater 3’UTR shortening in spermatogenesis. Genes are divided into five bins based on aUTR size, as indicated. The mean RED values between adjacent time points of all genes are plotted. *P* values (Wilcoxon rank-sum test) comparing gene bins 1 and 5 are shown. **c** Log2Ratio of RNAPII ChIP-seq levels between spermatids and pachynema (pachytene stage spermatocytes) for three groups of genes, namely 3’UTR shortened, unchanged and lengthened, around the TSS (+/− 1 kb, left), in gene body (middle) and around the last pA (+/− 1 kb, right). 3’UTR regulation was based on comparison of 4w and 2w samples. *P* values (K–S test) comparing genes with 3’UTR shortened (blue) or lengthened (red) with 3’UTR unchanged are indicated. **d** Log2Ratio of H3K4me3 ChIP-seq levels between spermatids and spermatocytes for three groups of genes, namely 3’UTR shortened, unchanged and lengthened, around the TSS (+/− 1 kb, left), in gene body (middle) and around the last pA (+/− 1 kb, right). 3’UTR regulation was based on comparison of 4w and 2w samples. *P* values (K–S test) comparing genes with 3’UTR shortened (blue) or lengthened (red) with 3’UTR unchanged are indicated

We next asked whether the regulated 3’UTR-APA sites had certain sequence and genomic features different from other sites. Interestingly, we noticed that genes with shortened 3’UTRs tended to have a significantly shorter cUTR and a longer aUTR than those with lengthened or unchanged 3’UTRs (*P* <1 × 10^−15^, K–S test, Fig. 4a). In addition, by dividing genes into five groups based on aUTR size (Fig. 4b), we found that the longer the aUTR the greater the extent of 3’UTR shortening, as indicated by the mean RED values of all genes. This trend was particularly noticeable for the 4w vs. 3w comparison (Fig. 4b), with *P* = 1.3 × 10^−41^ (Wilcoxon rank-sum test) for the difference between gene bins 1 (with the shortest aUTRs) and 5 (with the longest aUTRs). Since the aUTR size is relevant to competition between two 3’UTR pAs for usage [26], this result suggests that the 3’ end processing activity is generally regulated in spermatogenesis. Previous studies showed that the 3’UTR length inversely correlates with expression of proliferation genes [21] and C/P-related factors [20]. However, no obvious global change of expression was observed with either group in spermatogenesis (Additional file 1: Figure S1), suggesting that *trans* factor expression as a whole may not be the reason for APA regulation.

We next examined cis elements near the regulated pAs. Because proximal and distal pAs are surrounded by distinct cis elements [9, 52], we analyzed proximal and distal pAs separately (Additional file 1: Figure S2A). As such, upregulated proximal pAs of genes with shortened 3’UTR were compared with proximal pAs of other genes, and so were downregulated distal pAs. Overall, very few 4-mers were found to be significantly enriched in the −40 to +100 nt region around the regulated pAs (Additional file 1: Figure S2B). The top ones were UUGU and UUUU in the −40 to −1 nt region and the +1 to +100 nt region of distal pAs, respectively (Additional file 1: Figure S2B). More significant 4-mers were found in the −100 to −41 nt regions of proximal pAs, such as CGAC, AAGA and CACC, and of distal pAs, such as UAUU, UUUU and AUUU (Additional file 1: Figure S2B). However, their significance of enrichment is substantially lower than what we previously observed with APA regulation by C/P factors [26], and they appear to be related to mRNA stability regulation (see below). Thus, while it remains possible that some cis elements near the pA may regulate pA usage through binding to certain RBPs, this result does not support a global, cis element-based APA mechanism in spermatogenesis.

Prompted by the correlation between upregulation of gene expression and 3’UTR shortening (Fig. 3), we next asked whether APA changes in spermatogenesis were related to transcriptional regulation, as we previously observed across different tissue types and cell conditions [53]. To this end, we analyzed a chromatin immunoprecipitation and deep sequencing (ChIP-seq) data set for RNA polymerase II (RNAPII) [54], which included data for spermatids and pachytene stage spermatocytes (Additional file 1: Figure S3A). We found that genes with shortened 3’UTRs tended to have significantly higher RNAPII signals around the transcription start site (TSS), in the gene body and around the last pA, as compared to genes without 3’UTR-APA changes (*P* = 2.6 × 10^−9^, 2.0 × 10^−12^ and 5.7 × 10^−5^, respectively, K–S test, Fig. 4c). This result indicates that 3’UTR shortening is coupled with transcriptional upregulation in spermatogenesis.

We next asked whether chromatin structure, which is substantially remodeled in spermatogenesis and has been implicated in regulation of RNAPII activities [30], was related to 3’UTR-APA changes. To this end, we analyzed a ChIP-seq data set for H3K4 tri-methylation (H3K4me3) levels in spermatids and spermatocytes [32]. As expected, H3K4me3 levels were high around the TSS and downstream of the last pA (Additional file 1: Figure S3B). Importantly, the H3K4me3 level was significantly higher in spermatids vs. spermatocytes for genes with significant 3’UTR shortening than other genes (Fig. 4d) around the TSS, in the gene body and around the last pA (*P* = 7.3 × 10^−12^, 5.8 × 10^−63^ and 4.7 × 10^−8^, respectively, K–S test), indicating that APA regulation correlates with the H3K4me3 level. Since high H3K4me3 levels represent open chromatin state, this result suggests that chromatin structure change may lead to more efficient 3’ end processing, resulting in preferential usage of proximal pAs in 3’UTRs.

### 3’UTR shortening eliminates destabilizing elements that are highly potent in spermatogenesis

**Fig. 5:**
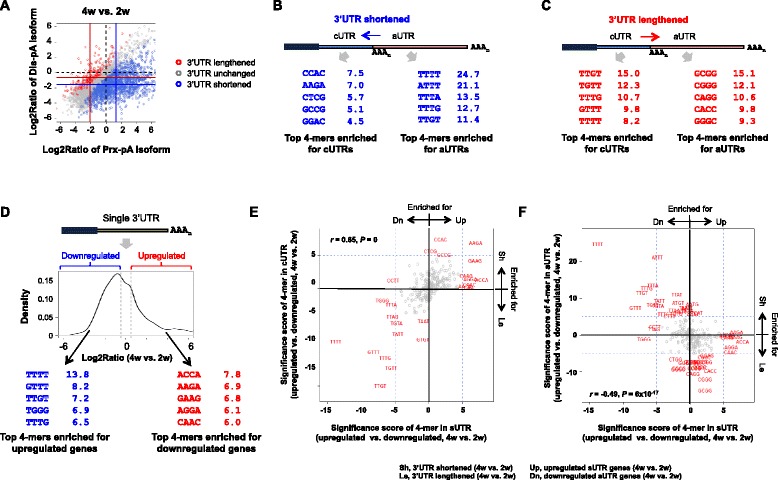
3’UTR cis elements contribute to mRNA abundance changes and APA profiles in spermatogenesis. **a** Scatterplot showing genes with lengthened, shortened and unchanged 3’UTRs between 4w and 2w samples. Significant 3’UTR-APA events were based on the SAAP analysis (FDR = 5 %) using the top two most abundant 3’UTR-pA isoforms of each gene. **b** Significant 4-mers enriched in cUTRs and aUTRs of genes with shortened 3’UTRs. Values are − log_10_(*P*), where *P* was based on the Fisher’s exact test examining the enrichment of 4-mers in cUTR (left) or aUTR (right) regions of genes with shortened 3’UTRs (4w vs. 2w). **c** As in (**b**) except that genes with lengthened 3’UTRs were analyzed. **d** Top, schematic showing cis element analysis using genes with only one pA, i.e., having a single 3’UTR (sUTR). Middle, upregulated and downregulated sUTR genes in the 4w vs. 2w comparison, corresponding to >1.4 fold change in RPM value, were selected for 3’UTR analysis. Bottom, top enriched 4-mers for sUTRs of downregulated genes (left) or upregulated genes (right). As in (**b**), values are − log10(*P*), where *P* was based on the Fisher’s exact test examining 4-mer enrichment. **e** Comparison of 4-mer enrichment in cUTR as calculated in (**b**) and (**c**) with that in sUTR as calculated in (**d**). X-axis values are − log_10_(*P*)*S, where *P* is the 4-mer enrichment *P* value for upregulated vs. downregulated genes, and S is 1 if a 4-mer is more enriched for sUTRs of upregulated genes, or −1 otherwise. Y-axis values are − log_10_(*P*)*S, where *P* is for enrichment of 4-mer in cUTRs of shortened or lengthened 3’UTRs, whichever is greater, and S is 1 if a 4-mer is more enriched for cUTRs of shortened 3’UTRs or −1 otherwise. **f** As in (**e**) except that y-axis is for enrichment of 4-mers in aUTRs of shortened or lengthened 3’UTRs

Because 3’UTR is important for mRNA metabolism, we next wanted to examine how 3’UTR shortening in spermatogenesis impacts cis elements in 3’UTRs. To this end, we first examined cis elements in shortened and lengthened 3’UTRs. To simplify analysis, we focused on two most abundant 3’UTR isoforms per gene and examined cis elements in cUTRs and aUTRs. As such, for each gene, the short isoform contained cUTR and the long isoform contained both cUTR and aUTR. Using APA events regulated between 4w and 2w samples (Fig. 5a) and 4-mers as indicators of cis elements, we found significant enrichments of U-rich, AU-rich and UG-rich elements in the aUTRs of shortened 3’UTRs, such as UUUU, AUUUU, UUUA, UUUG and UUGU (Fig. 5b). By contrast, the significance of 4-mers enriched in cUTRs of shortened 3’UTRs was rather modest, with the top 4-mers being CCAC, AAGA, CUCG, GCCG and GGAC (Fig. 5b). Interestingly, the cUTRs and aUTRs of lengthened 3’UTRs were also enriched with different 4-mers, including UG-rich and U-rich elements for cUTRs, such as UUGU, UGUU, UUUG, GUUU and UUUU; and G-rich elements, such as GCGG, CGGG and CAGG, for aUTRs (Fig. 5c). These results indicate that cis elements are highly biased in different portions of regulated 3’UTRs, suggesting potential impacts of 3’UTR cis elements on the APA profile.

To address if 3’UTR cis elements can regulate mRNA abundance in spermatogenesis, we selected genes with a single 3’UTR, or sUTR. As such, their expression could not be affected by APA. Interestingly, U-rich and UG-rich elements, such as UUUU, GUUU and UUGU, were significantly enriched in the sUTRs of downregulated genes (4w vs. 2w, Fig. 5d), indicating that these elements correlate with transcript abundance changes. Since these elements have previously been shown to play roles in mRNA stability [55, 56], it is likely that there exist potent mechanisms during spermatogenesis that degrade mRNAs containing these elements.

We next used the significance score (SS, derived from *P* value from the Fisher’s exact test, see Methods for detail) for each 4-mer to indicate its significance of enrichment in one set of sequences vs. another. We found that the SS values of 4-mers for sUTR regulation were positively correlated with those derived from cUTR analysis (*r* = 0.65, Pearson correlation, Fig. 5e), i.e., those associated with downregulated sUTR genes were also enriched in cUTRs of lengthened 3’UTRs, and those with upregulated sUTR genes were also enriched in cUTRs of shortened 3’UTRs (top 4-mers were highlighted in red in Fig. 5e). By contrast, a negative correlation was observed between the SS values of 4-mers associated with sUTR gene expression and those derived from aUTR analysis (*r* = −0.49, Pearson correlation, Fig. 5f). Overall, U-rich UG-rich and UA-rich 4-mers were highly enriched for sUTRs of downregulated genes, cUTRs of lengthened genes and aUTRs of shortened genes (Fig. 5e and f). It is also noteworthy that analyses using 6-mers gave very similar results (Additional file 1: Figure S4). Taken together, these results indicate that cis elements in 3’UTRs play a significant role in regulating mRNA abundance in spermatogenesis, likely through control of mRNA stability, and hence contribute to the APA profiles. Conversely, our data also suggest that shortening of 3’UTR can significantly remove destabilizing elements, impacting gene expression.

### 3’UTR shortening substantially removes transposable elements in the transcriptome

**Fig. 6:**
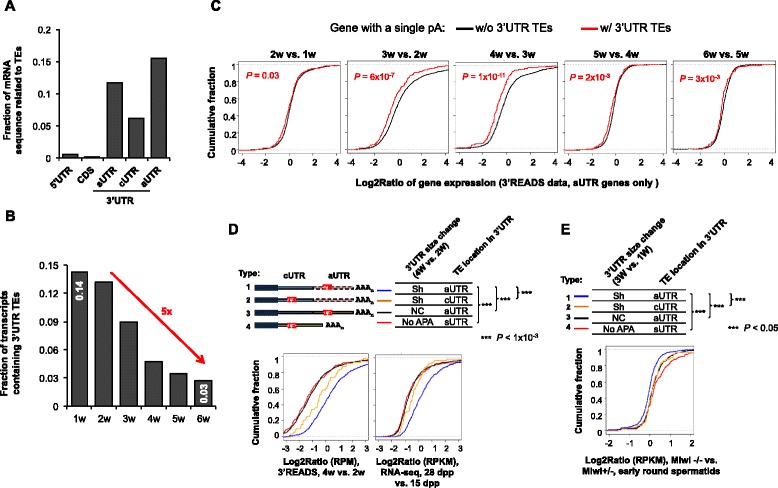
3’UTR shortening eliminates TEs. **a** TE contents in 5’UTR, CDS and 3’UTR of all genes. aUTR, alternative 3’UTR; cUTR, common 3’UTR; sUTR, single 3’UTR (gene without APA). The fraction of mRNA sequence related to TEs was based on the number of nucleotides of the TEs annotated by the RepeatMasker track of UCSC Genome Browser (mm9). **b** Change of TE-containing mRNAs in spermatogenesis. The fraction of the transcripts containing 3’UTR TEs at each time point is plotted. **c** Gene expression changes for genes with or without 3’UTR TEs. Only genes with a single 3’UTR (sUTR) were used for this analysis. Expression was based on the 3’READS data. *P* values (K–S test) indicating difference between the two gene groups are shown. **d** Expression changes of four types of genes. A type 1 gene has 3’UTR shortened and contains TEs only in aUTR; a type 2 gene also has 3’UTR shortened but contains TEs in cUTR only; a type 3 gene has 3’UTR unchanged and contains TEs in aUTR; a type 4 gene has a single 3’UTR (no APA) and there are TEs in the 3’UTR. All APA regulation was based on the 4w vs. 2w comparison. Bottom, gene expression was analyzed using 3’READS data (4w vs. 2w, left), or CDS reads of RNA-seq data (28 dpp vs. 15 dpp, right). *P* values (K–S test) indicating difference between type 1 genes and others are indicated on the top. **e** Gene expression difference in Miwi−/− vs. Miwi+/− at the early round spermatid stage for the four gene types described in (**d**) except that the APA analysis was based on 3w vs. 1w comparison. Gene expression was based on RNA-seq reads mapped to CDS. The type 1 gene set was compared with other types using the K–S test. *P* values are all significant (<1 × 10^−3^)

Recent studies have indicated that pachytene piRNAs can degrade target mRNAs through imperfect base-pairing [45]. This mechanism has been implicated in degradation of transcripts containing transposable elements (TEs). We found that TE sequences detected by the RepBase database [57, 58] accounted for a sizable fraction of the 3’UTR repertoire in the mouse genome: for genes with a single pA, TEs accounted for 12 % of their 3’UTRs; for genes with APA, TEs accounted for 6 % and 16 % of their cUTR and aUTR sequences, respectively (Fig. 6a). Consistently, 3’UTR shortening in spermatogenesis led to ~5-fold decrease of the total number of transcripts with TEs (Fig. 6b). Using genes with a single pA, we found TEs in 3’UTRs could lead to significant downregulation of mRNA transcripts between 3w and 2w (*P* = 6 × 10^−7^, K–S test) and between 4w and 3w (*P* = 1 × 10^−11^, K–S test) (Fig. 6c), confirming the negative impact of 3’UTR TEs on mRNA expression.

To examine how 3’UTR shortening impacts TE-mediated mRNA degradation, we divided genes into four groups based on both 3’UTR regulation between 4w and 2w and TE location in the 3’UTR (Fig. 6d, top): 1) genes with 3’UTRs shortened and with TEs in aUTR (TEs can be eliminated by 3’UTR shortening); 2) genes with 3’UTRs shortened and with TEs in cUTR (TEs cannot be eliminated despite 3’UTR shortening); 3) genes with unchanged 3’UTR and with TEs in aUTR (no APA regulation and TEs cannot be eliminated); 4) genes with a single 3’UTR which contains TEs (no APA and TEs cannot be eliminated). Both 3’READS data and RNA-seq data showed that genes with shortened 3’UTRs and with TEs in aUTR (group 1) tended to have significantly higher expression levels than genes in other groups between 4w and 2w (*P* <1 × 10^−3^, K–S test, Fig. 6d, bottom). This result suggests that elimination of TEs in 3’UTRs by 3’UTR shortening leads to higher transcript abundance.

To further address whether the TE-mediated gene regulation is due to mRNA degradation by the piRNA/Miwi pathway, we analyzed RNA-seq data from Miwi−/− and Miwi−/+ mice [44]. Focusing on the four groups of genes described above, we found that group 1 genes were significantly less activated in Miwi−/− vs. Miwi−/+ than the other three groups of genes (*P* <0.05, K–S test, Fig. 6e), indicating that downregulation of gene groups 2–4 in wild type mice were mediated by Miwi. This result further supports the notion that 3’UTR shortening helps avoid TE/piRNA/Miwi-mediated mRNA degradation in spermatogenesis. It is also notable that TE regulation was independent of the U-rich elements described above, because U-rich elements could still be detected after excluding TE sequences in analysis (Additional file 1: Figure S5A) and TE-mediated regulation identified by the Miwi−/− vs. Miwi−/+ comparison did not involve U-rich elements (Additional file 1: Figure S5B).

### Widespread regulation of C/P events in introns

**Fig. 7:**
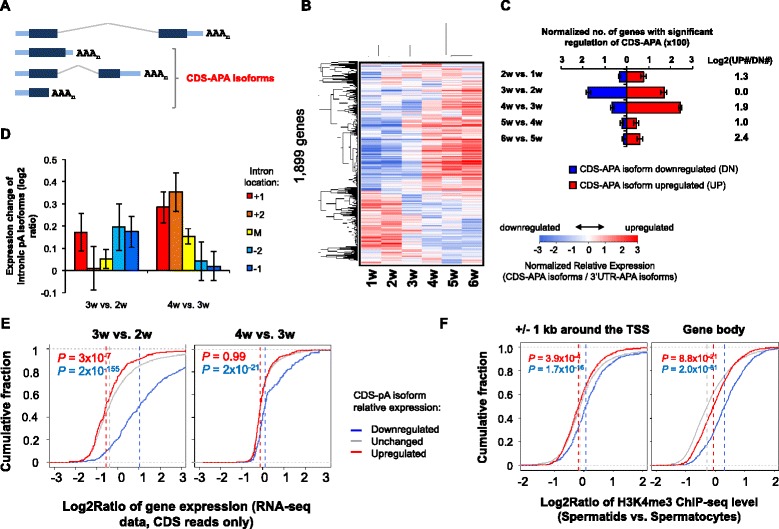
Regulation of CDS-APA in spermatogenesis. **a** Schematic of CDS-APA. CDS-APA isoforms are those using pAs in introns or non-3’-most exons. **b** Heatmap showing relative expression (RE) values of CDS-APA isoforms vs. 3’-most exon isoforms. RE values are mean-centered and clustered using hierarchical clustering with Pearson correlation coefficient as metric. **c** Normalized number of genes with significant regulation of CDS-APA as identified by GAAP and SAAP analyses (FDR = 5 %) (see Methods for details). The ratio of number of genes with upregulated (UP) CDS-APA isoforms to that with downregulated (DN) isoforms is indicated. **d** Regulation of isoforms using pAs in different introns. Introns were divided into five groups, i.e., the first and second introns (+1 and +2, respectively), the last and second to last introns (−1 and −2, respectively) and middle introns (M). The expression change of isoform is based on RPM values. **e** Gene expression changes vs. CDS-pA regulation. Genes were divided into three indicated groups based on CDS-APA regulation between comparing samples (SAAP analysis, FDR = 5 %). The median value for each group is indicated by a dotted vertical line. The *P* value (K–S test) for difference between genes with upregulated or downregulated CDS-APA and those with unchanged CDS-APA is shown in each graph (in red or blue, respectively). RNA-seq data with CDS reads were used for gene expression analysis. **f** ChIP-seq analysis of H3K4me3 levels on genes with CDS-APA regulation. Log2Ratio of ChIP-seq levels between spermatids and spermatocytes for the three gene groups is shown. CDS-APA regulation was based on comparison of 4w and 2w samples, corresponding to spermatid and spermatocyte stages, respectively. *P* value (K–S test) comparing genes having downregulated (blue) or upregulated (red) CDS-pA isoforms with genes having CDS-APA unchanged is indicated

Over ~40 % of mouse genes express mRNA isoforms using pAs in introns or exons upstream of the 3’-most exon [12] (illustrated in Fig. 7a). These APA events are commonly called CDS-APA because they lead to APA isoforms with different CDS. Regulation of CDS-APA can be controlled by both splicing and C/P activities [26]. Comparing CDS-pA isoform expression with that of 3’UTR-APA isoforms, we found samples could be clustered into two groups (Fig. 7b), i.e., 1–3 weeks and 4–6 weeks, similar to using the 3’UTR-APA data (Fig. 1c). Using SAAP and GAAP analyses, we found that both 3w vs. 2w and 4w vs. 3w comparisons involved a large number of regulated CDS-APA events. Interestingly, while similar numbers of genes displayed upregulated and downregulated isoforms in 3w vs. 2w, a much greater number of genes showed upregulation of CDS-pA isoforms in 4w vs. 3w, indicating distinct mechanisms involved in CDS-APA in these two phases. Consistently, we found that intronic pAs close to the 5’ end of a gene were more likely to be activated in 4w vs. 3w (Fig. 7d), a trend not observed with 3w vs. 2w (Fig. 7d). In addition, gene groups regulated by CDS-APA in 4w vs. 3w were associated with distinct GO terms than those with CDS-APA regulated in 3w vs. 2w (Table 2). By contrast, other features, including intron size, 5’ splice site strength, or 3’ splice site strength, did not appear to be significantly associated with CDS-APA regulation at either stage (Additional file 1: Figure S6).

**Table 2:** Gene ontology terms associated with genes with regulated CDS-APA in spermatogenesis

−log_10_(*P*), downregulated CDS-APA		−log_10_(*P*), upregulated CDS-APA		Gene ontology terms
3w vs. 2w	4w vs. 3w	3w vs. 2w	4w vs. 3w	
Biological process				
12.6	0.6	0.6	1.4	spermatogenesis
6.2	0.4	0.4	0.7	cilium movement
5.7	1.4	1.4	0.3	cellular process involved in reproduction in multicellular organism
5.2	0.5	0.5	0.6	fertilization
5.0	0.8	0.8	1.3	cilium organization
Cellular component				
7.5	0.5	1.5	0.4	cilium
0.4	1.5	0.4	5.2	intracellular non-membrane-bounded organelle
1.0	0.0	1.3	4.7	Golgi apparatus
4.3	0.1	2.1	0.2	sperm part
4.2	2.1	0.2	0.1	ciliary cytoplasm

*P* is based on the Fisher’s exact test. Only top five terms for each of the two categories, based on the maximum *P* value across all comparisons, are shown

Compared to genes with unchanged CDS-APA, genes with downregulated CDS-APA tended to be significantly upregulated (*P* = 2 × 10^−155^ and 2 × 10^−21^ for 3w vs. 2w and 4w vs. 3w, respectively, K–S test, Fig. 7e), whereas genes with upregulated CDS-APA tended to be either mildly downregulated in 3w vs. 2w, or unchanged in 4w vs. 3w (Fig. 7e). In addition, genes with downregulated CDS-APA tended to have increased H3K4me3 signals around the TSS and in the gene body (Fig. 7f), whereas those with upregulated CDS-APA tended to have mildly decreased levels, as compared to genes with unchanged CDS-APA (Fig. 7f). Thus, the usage of upstream pAs appears to be inhibited when gene expression is upregulated and chromatin becomes more open, a trend that is opposite to the usage of proximal pA in 3’UTRs (see above). Whether this difference is due to the involvement of splicing activity, which plays a major role in CDS-APA [26], remains to be examined in the future.

### Significant activation of bidirectional transcription in spermatogenesis

**Fig. 8:**
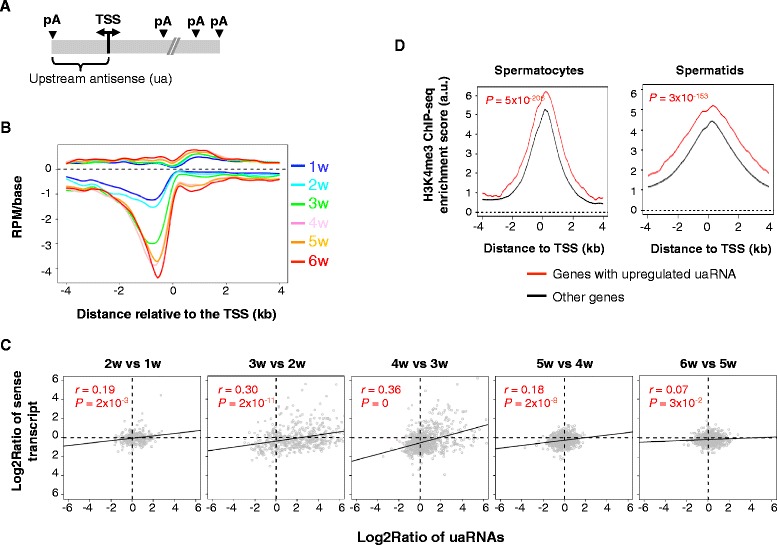
Widespread activation of upstream antisense RNA expression in spermatogenesis. **a** Schematic of upstream antisense RNA (uaRNA). An uaRNA is defined as an antisense transcript with pA located within 2 kb from the transcription start site (TSS) of a gene. **b** uaRNA expression is significantly upregulated in spermatogenesis. 3’READS data were used for the plot. Reads per million (RPM) value was assigned to each pA location. **c** Relationship between uaRNA expression and sense RNA expression at different time points. Correlation is indicated by the Pearson correlation coefficient (*r*) and *P* value for linear regression. **d** ChIP-seq analysis of H3K4me3 levels on genes with uaRNA regulation. Enrichment scores of H3K4me3 +/− 4 kb around the TSS are plotted for genes with upregulated uaRNAs or other genes. *P* values were based on the Wilcoxon rank-sum test comparing RPM values of the two gene groups in the +/− 1 kb region from the TSS

A large fraction of RNAPII promoters in mammalian cells are bidirectional, leading to both sense and antisense transcripts (reviewed in [59, 60] and illustrated in Fig. 8a). The antisense transcripts upstream of the TSS are typically called PROMPTs or uaRNAs. Recent studies have indicated that C/P is involved in termination of uaRNAs [28, 29], and PABPN1 and the exosome complex regulate their abundance [26]. Consistent with our previous data using mouse C2C12 cells [26], we found uaRNA pAs in testis samples were widely distributed within 2 kb from the TSS, peaking around −700 nt (Fig. 8b). Importantly, their expression was significantly upregulated during spermatogenesis, with the most significant phase for upregulation being 3w vs. 2w (Fig. 8b). We found that there was a general correlation between uaRNA expression changes and regulation of their sense strand transcripts (Fig. 8c), with *r* = 0.30 and *r* = 0.36 (Pearson correlation) for 3w vs. 2w and 4w vs. 3w, respectively. Further analysis of the H3K4me3 ChIP-seq data showed that the TSS regions of genes with upregulated uaRNAs had much higher H3K4me3 signals in both spermatocytes and spermatids (*P* = 5 × 10^−206^ and 3 × 10^−153^, respectively, Wilcoxon rank-sum test). However no difference could be discerned between spermatocytes and spermatids (data not shown). This result indicates that open chromatin may be a prerequisite for uaRNA regulation, but the extent of its regulation is governed by other factors, possibly the activity of the corresponding bidirectional promoter.

## Discussion

Here we show widespread shortening of 3’UTR by APA in spermatogenesis, with 3’UTRs being the shortest in spermatids. While this mechanism appears to be general, some genes tend to be regulated to a greater extent than others. Testis-specific genes display more substantial 3’UTR shortening than ubiquitously expressed genes, highlighting the importance of this mechanism for spermatogenesis. Interestingly, protein ubiquitination is the most significant pathway associated with genes with 3’UTR shortening. Because of the importance of ubiquitination for sperm development after spermatids, such as nucleosome removal and remodeling of cell structure, we posit that 3’UTR shortening plays an important role for spermiogenesis. Further experimental studies are needed to confirm this hypothesis.

Using genes without APA as a control group, we reveal that 3’UTR elements play an important role in mRNA abundance during spermatogenesis, including U-rich, UA-rich and UG-rich elements, possibly through regulation of mRNA stability. This may be important for elimination of mRNAs before spermatozoa, which contain little mRNA [61]. Future studies are needed to elucidate proteins involved in the mRNA degradation mediated by these cis elements. Another important question to be addressed is how much of a role APA plays to help transcripts evade degradation. Compared to sUTR transcripts, long 3’UTR isoforms decreased in expression by ~1.7-fold from 2 weeks to 4 weeks and short isoforms increased by about 2.1-fold in the same period (Additional file 1: Figure S7). Assuming long isoforms have similar decay rates to sUTR transcripts, their decrease in expression can be attributable to APA changes. Therefore, while future experiments are needed to precisely address the question, our analysis indicates a significant impact on mRNA stability through APA changes. In the same vein, our cis element analysis result can parsimoniously explain why some genes display 3’UTR lengthening while the global trend is shortening: for genes whose cUTRs are enriched with destabilizing elements, their short 3’UTR isoforms are less stable than the long isoform, which presumably contains additional stabilizing elements in the aUTR to negate the destabilizing effect of cUTR, leading to overall display of 3’UTR lengthening.

By analyzing APA isoforms containing TEs at different portions of 3’UTR, we corroborated the recent finding by Gou et al. [45] that 3’UTR TEs are targeted for degradation in spermatogenesis. Using gene expression data from Miwi−/− mice [44], we further found that the degradation is through the piRNA-Miwi pathway. Thus, 3’UTR shortening can help genes evade the TE/piRNA/Miwi-based mRNA elimination during spermatogenesis. Previous studies have shown that TEs in 3’UTRs can play regulatory roles for mRNA metabolism [62–64] and some TEs can confer functional pAs to the host gene [65]. APA regulation in spermatogenesis can thus effectively permit evolution of TEs in 3’UTRs without inhibiting the expression of host genes, contributing to exaptation of TEs into 3’UTRs. Further studies are needed to elucidate how important this mechanism is for 3’UTR evolution. Also to be examined is whether some TE-containing aUTR sequences can give rise to piRNAs, which in turn regulate the host gene post-transcriptionally.

We found that 3’UTR shortening is coupled with upregulation of gene transcription and open state of chromatin, as indicated by RNAPII and H3K4me3 levels, respectively. Open chromatin has been suggested to cause widespread transcription in testis [30], leading to high complexity of the transcriptome. While this result is consistent with our previous finding implicating a role of transcriptional activity in APA regulation [53], the mechanism behind the coupling is unclear. One possibility is that permissive chromatin structure makes it more efficient to assemble the cleavage/polyadenylation machinery, leading to more usage of proximal pAs. However, other mechanisms involving specific factors to facilitate recruitment of the C/P machinery, such as that mediated by transcription factors [66], cannot be ruled out.

Widespread regulation of APA events in introns and internal exons suggests modulation of splicing activity during spermatogenesis, which is consistent with previous reports [31, 34–37]. Notably, the CDS-APA regulation between 3 and 4 weeks is reminiscent of the APA regulation by U1 snRNP inhibition [26, 27], where activation of pAs is largely biased to 5’ introns. Whether there is a localized shortage of U1 snRNP for certain genes leading to activation of intronic pAs needs to be examined in the future. Interestingly, genes with suppressed CDS-APA isoforms tend to be upregulated in expression (Fig. 7e) and have higher H3K4me3 levels (Fig. 7f), suggesting that open chromatin state may lead to efficient splicing, resulting in inhibition of intronic C/P.

We found global activation of uaRNAs during spermatogenesis (Fig. 8). These non-coding transcripts are generated by divergent promoters and are typically under the surveillance of the exosome [26, 29]. Their expression can significantly enrich the transcriptome during spermatogenesis, potentially impacting evolution of new genes [67]. uaRNA expression appears to be associated with open chromatin around the TSS and correlates with the expression of sense transcripts. It is not clear, however, why they are not eliminated by the nuclear exosome. Whether the function of exosome is suppressed in spermatogenesis or is overwhelmed by substantial activation of uaRNA expression needs to be addressed in the future.

## Conclusions

**Fig. 9:**
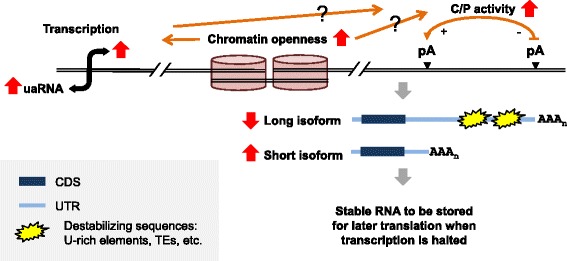
A proposed model for the mechanism and consequence of APA regulation in spermatogenesis. During maturation of spermatocytes into spermatids, chromatin becomes more open, leading to more transcriptional activities. Open chromatin and/or heightened transcription may create a more permissive cis environment for cleavage/polyadenylation (C/P). The enhanced C/P activity results in more usage of proximal pAs, leading to higher expression of short 3’UTR isoforms relative to long isoforms. Isoforms with short 3’UTRs avoid destabilizing cis elements in 3’UTRs, such as U-rich elements and TEs, which are potent during spermatogenesis. Stable isoforms are stored for translation after the spermatid stage when transcription is globally halted. Question marks indicate detailed mechanism(s) are not clear

Here we show widespread shortening of 3’UTR by APA in spermatogenesis, with 3’UTRs being the shortest in spermatids. By combining our data with other data, including RNA-seq, RNAPII binding and H3K4me3 signals, we propose a model which explains the mechanism of APA regulation and its consequence (Fig. 9): open chromatin in spermatogenesis may create a favorable cis environment for 3’ end processing, possibly by allowing more efficient assembly of the C/P complex. Enhanced C/P leads to preferential usage of proximal pAs, resulting in shortened 3’UTRs. Transcripts with a short 3’UTR can evade powerful mRNA degradation mechanisms in play during spermatogenesis. Stabilized transcripts, whose protein products are enriched with functions important for sperm maturation, can be efficiently stored and translated after the spermatid stage when transcription is halted. In sum, APA in spermatogenesis connects regulation of chromatin status with post-transcriptional control, impacting sperm maturation.

## Methods

### 3’READS of testis samples

Testis samples were obtained from mice (FVB) by surgical removal according to the approved Institutional Animal Care and Use Committee (IACUC) protocols. The 3’ region extraction and deep sequencing (3’READS) method was previously described in [68]. Briefly, 25 μg of input RNA was used for each sample, and poly(A) + RNA was selected using oligo d(T)25 magnetic beads (NEB, Ipswich, MA, USA), followed by on-bead fragmentation using RNase III (NEB). Poly(A) + RNA fragments were then selected using the chimeric U_5_ and T_45_ (CU_5_T_45_) oligo conjugated on streptavidin beads, followed by RNase H (NEB) digestion. Eluted RNA fragments were ligated with 5’ and 3’ adapters, followed by RT and PCR (15x) to obtain cDNA libraries for sequencing on the Illumina platform. Processing of 3’READS data was carried out as previously described [68]. Briefly, reads were mapped to the mouse genome using Bowtie 2 (Langmead and Salzberg 2012). Reads with ≥2 unaligned Ts at the 5’ end are called poly(A) site-supporting (PASS) reads, which were used to identify pAs. pAs located within 24 nt from each other were clustered together. The number of PASS reads generated for each sample is listed in Additional file 1: Table S2.

### APA analysis

Relative expression (RE) of two 3’UTR-APA isoforms, e.g., Dis-pA and Prx-pA, was calculated by log2(RPM_Dis-pA_/RPM_Prx-pA_), where RPM was reads per million PASS reads. Relative expression difference (RED) of two isoforms in two comparing samples was based on difference in RE of the two isoforms in the two samples. For CDS-APA analysis, RE was based on comparison of all CDS-pA isoforms combined (CDS-pA set) with all 3’UTR-pA isoforms combined (3’UTR-pA set), and RED was also based on the two sets. We used the significance analysis of alternative polyadenylation (SAAP) method to identify significantly regulated APA events, as previously described [26]. Briefly, for two pAs (or two pA sets) from two comparing samples, a RED score was first calculated and was called observed RED. The PASS reads were then sampled based on the assumption that the relative abundance of each pA isoform was the same in two samples. Sampling was performed 20 times to obtain expected mean and standard deviation of RED, which were then used to convert observed RED to Z score (minus mean and divided by standard deviation). False discovery rate (FDR) was calculated by comparing observed Z (Zo) and expected Z (Ze) for a given Z cutoff value (Zc). For 3’UTR-APA, we selected the two most abundant pA isoforms for analysis. For CDS-APA, we combined all isoforms using pAs in upstream regions of the 3’-most exon and compared their expression change with that of isoforms using pAs in the 3’-most exon. Individual CDS-pAs were also analyzed by comparing to all other pA isoforms of the gene. For uaRNA analysis, we combined all antisense transcripts using pAs within 2 kb upstream from the transcription start site (TSS), excluding those mapped to other mRNA genes, and compared them to all sense strand transcripts, excluding pAs located within 2 kb downstream of the TSS. We used FDR = 5 % to select significantly regulated APA events. Global analysis of alternative polyadenylation (GAAP) was previously described in [26]. Briefly, for two 3’READS data sets A and B, we sampled by bootstrapping 1.5 M PASS reads from A and B, respectively. The number of genes with significant APA changes based on SAAP analysis was calculated and called observed value. The data were also randomly permutated (shuffled across samples) to obtain the expected value. The observed value − expected value was normalized number of genes with significant APA regulation. This process was repeated 20 times to obtain standard deviation. The weighted mean of 3’UTR size for each gene was based on 3’UTR sizes of all 3’UTR-APA isoforms, weighted by the expression level of each isoform based on the number of PASS reads. Only genes with ≥50 PASS reads were used for the analysis.

### Cis element analysis

To identify cis elements enriched for a set of sequences, we compared k-mer (k = 4 or 6) frequencies in the set vs. a background set and derived *P* values indicating significance of enrichment using the Fisher’s exact test. This was carried out to identify enriched k-mers for cUTR, aUTR and sUTR sets. For cis elements around regulated pAs, we first put proximal and distal pAs into two separate sets to mitigate the possibility that identified cis elements were related to location, rather than regulation. Regulated proximal pAs were then compared to other proximal pAs to identify overrepresented k-mers. The same approach was used for distal pAs. We examined three regions around the pA, i.e., −100 to −41 nt, −40 to −1 nt and +1 to +100 nt. For each region, the Fisher’s exact test was used to examine whether a k-mer was enriched for a set of pAs vs. other pAs.

### Analysis of introns

The intron location was based on the RefSeq database, considering all RefSeq-supported splicing isoforms. Distribution of regulated intronic pA isoforms was compared to that of background set, which was derived from all detected intronic pAs in the mouse genome [12]. To calculate 5’ splice site (5’SS) or 3’ splice site (3’SS) strength, we used all 5’SS or 3’SS supported by mouse RefSeq sequences and applied MaxEntScan to obtain strength scores [69]. The 5’SS or 3’SS strength of introns containing regulated pAs was compared to that of background introns with the same relative locations in genes by the Wilcoxon rank-sum test.

### Other analyses

Gene expression using 3’READS data was based on all PASS reads mapped to the 3’-most exon of a gene, represented by the reads per million total PASS reads (RPM) value. For RNA-seq data analysis, reads were mapped to the mouse genome (mm9) using Bowtie 2 (local mode). Only uniquely mapped reads with MAPQ score >10 were used. RefSeq gene models were used to calculate gene expression level. In order to eliminate the effect of 3’UTR-APA on gene expression calculation, only reads mapped to CDS were used. For ChIP-seq data analysis, reads were mapped to the mouse genome (mm9) using Bowtie 2. Only uniquely mapped reads with MAPQ score >10 were used. Reads mapped to the same genomic loci (defined by chromosome, strand and start and end positions) were collapsed into one. The genomic regions were binned for enrichment score calculation, i.e., every 50-nt before the TSS and after the last pA, or every percentile of the gene body. The enrichment score was defined as log2(ratio) of reads per million total mapped reads (RPM) of immunoprecipitated sample to that of input sample. The 5’ end position of a read was used to represent the read. For TE content analysis, 5’UTR and CDS were defined by RefSeq annotations, and 3’UTR was based on both RefSeq annotations and 3’READS data. All TE types were analyzed, including LINE, SINE, LTR and DNA. Gene ontology (GO) analysis was carried out using the Fisher’s exact test. GO annotation of genes was obtained from the NCBI Gene database. Testis-specific genes and ubiquitously expressed genes were defined in [70]. The following data sets were downloaded from the GEO database of NCBI: [NCBI GEO:GSE45441] (RNAPII ChIP-seq data); [NCBI GEO:GSE49621] (H3K4me3 ChIP-seq data); [NCBI GEO:GSE43717] (directional RNA-seq data for different cell types in testis); [NCBI GEO:GSE42004] (directional RNA-seq data for different stages of testis from Miwi +/− and Miwi−/− strains).

### Availability of supporting data

All data can be obtained from the NCBI GEO database [NCBI GEO:GSE73973].

## Additional file

Additional file 1: Figure S1. Gene expression changes of proliferation genes and C/P-related genes. **Figure S2.** Analysis of cis elements around pAs of shortened 3’UTRs. **Figure S3.** ChIP-seq data for RNAPII and H3K4me3 in spermatids and spermatocytes. **Figure S4.** 3’UTR cis elements contribute to APA profiles in spermatogenesis. **Figure S5.** The effect of 3’UTR TEs on gene expression is independent of U-rich elements. **Figure S6.** Features of the introns containing regulated pAs. **Figure S7.** Transcript expression changes from 2 weeks to 4 weeks. **Table S1. **Top five more significant Ingenuity Canonical Pathways enriched for genes with shortened 3’UTRs as determined by the Ingenuity Pathway Analysis. **Table S2.** Statistics of 3’READS data. (PDF 625 kb)
