# Supplementary material for: Alternative cleavage and polyadenylation in spermatogenesis connects chromatin regulation with post-transcriptional control
Source: BMC Biol. 2016 Jan 22;14:6. doi: 10.1186/s12915-016-0229-6 (PMC4724118; doi:10.1186/s12915-016-0229-6)
Supplement: Additional file 1: Figure S1. — Gene expression changes of proliferation genes and C/P-related genes. Figure S2. Analysis of cis elements around pAs of shortened 3’UTRs. Figure S3. ChIP-seq data for RNAPII and H3K4me3 in spermatids and spermatocytes. Figure S4. 3’UTR cis elements contribute to APA profiles in spermatogenesis. Figure S5. The effect of 3’UTR TEs on gene expression is independent of U-rich elements. Figure S6. Features of the introns containing regulated pAs. Figure S7. Transcript expression changes from 2 weeks to 4 weeks. Table S1. Top five more significant Ingenuity Canonical Pathways enriched for genes with shortened 3’UTRs as determined by the Ingenuity Pathway Analysis. Table S2. Statistics of 3’READS data. (PDF 625 kb) [file 12915_2016_229_MOESM1_ESM.pdf]

SUPPLEMENTARY INFORMATION

Supplementary Figures

Figure S1. Gene expression changes of proliferation genes (left) and C/P-related genes (right). The proliferation gene set was based on those reported in [21] and the C/P-related gene set was based on those reported in [6]. P-value (Wilcoxon rank-sum test) comparing proliferation gene set or C/P-related gene set with other genes is indicated.

Figure S2. Analysis of cis elements around pAs of shortened 3'UTRs. (A) Schematic showing the cis element analysis strategy. Prx-pAs and Dis-pAs were compared within their respective groups of pAs from genes without APA changes to minimize the influence of location-associated cis elements. **(B)** Cis elements enriched for Prx-pAs (left) or Dis-pAs (right) of genes with shortened 3'UTRs. Three regions around the pA were analyzed, i.e., -100 to -41 nt, -40 to -1 nt, and +1 to +100 nt. The 4w vs. 2w comparison data were used.

Figure S3. ChIP-seq data for RNAPII and H3K4me3 in spermatids and spermatocytes. (A) ChIP-seq data (GSE45441, NCBI GEO database) for RNAPII distribution on genes in spermatids or pachynema (pachytene stage spermatocytes). The 3 kb region upstream of the transcription start site (TSS) and 3 kb region downstream of the 3'-most pA are shown with absolute distance values (kb), and the region between TSS and 3'-most pA, or gene body, is shown with relative values, with the whole region set to 100%. Enrichment score (y-axis) was calculated based on log2Ratio of reads per million (RPM) for the immunoprecipitated sample to that of input sample. **(B)** ChIP-seq data (GSE49621, NCBI GEO database) for H3K4me3 distribution on genes in spermatids or spermatocytes. The 3 kb region upstream of the transcription start site (TSS) and 3kb region downstream of the 3'-most pA are shown with absolute distance values (kb), and the region between TSS and 3'-most pA, or gene body, is shown with relative values, with the whole region set to 100%. Enrichment score (y-axis) was calculated based on log2Ratio of reads per million (RPM) for the immunoprecipitated sample to that of input sample.

Figure S4. 3'UTR cis elements contribute to APA profiles in spermatogenesis. (A) As in Figures 5B and 5C, significant 6-mers enriched in cUTRs and aUTRs of genes with shortened 3'UTRs (top) or lengthened 3'UTRs (bottom). **(B)** As in Figure 5D, top enriched 6-mers for sUTRs of downregulated genes (left) or upregulated genes (right).

Figure S5. The effect of 3'UTR TEs on gene expression is independent of U-rich elements. (A) Top 4-mers and 6-mers enriched in 3'UTRs of upregulated vs. downregulated genes in the 4w vs. 2w comparison, as in Figure 5D. To address the contribution of TEs, we used only non-TE sequences for this analysis. Values are $-\log_{10}(P)$, where *P* was based on the Fisher's exact test examining bias of 4-mer/6-mer frequency in the 3'UTRs of upregulated genes vs. downregulated genes. **(B)** Top 4-mers and 6-mers enriched in 3'UTRs of upregulated and downregulated genes in the Miwi-/- vs. Miwi+/- comparison. To eliminate the effect of 3'UTR changes on gene expression analysis, only genes with a single 3'UTR were used.

Figure S6. Features of the introns containing regulated pAs. Intron size, and 5' splice site (5'SS) and 3' splice site (3'SS) strengths of introns with upregulated intronic pAs **(A)** or with downregulated intronic pAs **(B)** were analyzed. Numbers are significance score (SS), which was calculated by $-\log_{10}(P)*S$, where *P* was based on the Wilcoxon rank-sum test comparing an intron set of interest with a background set, and S = 1 when the intron set of interest had a larger median value (intron size, 5'SS strength or 3'SS strength) than the background set or -1 otherwise. The background set was derived from all mouse introns containing pAs with detectable usage (see Materials and Methods). Introns were divided into five groups based on relative location, as in Figure 7D. The SS data are colored according to the color scheme shown in the graph.

Figure S7. Transcript expression changes from 2 week to 4 weeks. Transcripts are divided into three groups, i.e., sUTR transcripts, short 3'UTR transcripts (Prx-pA) and long 3'UTR transcripts (Dis-pA). Median values are shown. Difference between median values of sUTR transcripts and of short/long 3'UTR transcripts is indicated. P-value (K-S test) indicating the significance of difference is shown.

Supplementary Tables

Table S1. Top five more significant Ingenuity Canonical Pathways enriched for genes with shortened 3'UTRs as determined by the Ingenuity Pathway Analysis

Ingenuity Canonical Pathways	-log(*P*)	Molecules
Protein Ubiquitination Pathway	17.2	FZR1,USP45,UBE3B,UBR2,USP20,UBE2V2,HSPA5,SKP1,TCEB1,USP48,DNAJC28,HSPA4,UBE2B,BAG1,SUGT1,UBE2E3,BRCA1,DNAJC30,ANAPC11,USP36,DNAJC27,PSMD5,THOP1,USP31,UBE2G2,UBE2L3,DNAJC5,PSMD12,DNAJC18,DNAJC14,HSPA13,SMURF2,VHL,PSMD9,CUL1,ANAPC10,DNAJC10,USP2,UBE4A,SMURF1,USP3,USO1,PSMD10,HSPA12B,PSMC6,USP16,USP47,PSMA3,DNAJB1,AMFR,HSPA4L,HSPH1,UBE2R2,MDM2,PSMB6,DNAJB9,UBE3A,PSMD8,HSPA12A,DNAJC21,UBE2J1,UBE2E2,USP37,USP46,BTRC,USP49,DNAJC7,UBE2D3,USP25
IGF-1 Signaling	5.7	IGFBP4,AKT2,PXN,NRAS,MAPK1,YWHAE,PRKAR2A,SRF,YWHAZ,PDPK1,KRAS,GRB10,PIK3R3,PTK2,RRAS2,PRKCI,IGF1,MAP2K2,PRKAG2,MRAS,SOCS7,RASA1,SOCS5,PRKAR1A
UVC-Induced MAPK Signaling	5.1	NRAS,MAPK1,MAPK9,KRAS,BRAF,PRKCI,RRAS2,SMPD4,MAP2K2,PRKCD,MRAS,MAPK10,SMPD3,PRKCB
ATM Signaling	5.1	SMC3,CREB3,ABL1,CCNB2,MAPK9,MDM2,MRE11A,TDP1,CBX5,CDK1,ATF2,RAD51,SMC2,MAPK10,TP53BP1,TLK2,BRCA1
Huntington's Disease Signaling	5.1	MAPK1,VTI1A,PACSIN1,EGF,PDPK1,HSPA5,AP2A2,VTI1B,GNB1,HSPA4,CDK5,IGF1,HDAC11,VAMP3,DNAJB1,GOSR1,GNB1L,RASA1,NAPA,SDHA,AKT2,MAP2K7,PSMF1,SH3GL3,CREB3,TBP,RCOR1,MAPK9,HIP1,PSME3,ATF2,TAF9B,PIK3R3,PLCB4,PRKCI,DNAJC5,PRKCD,CAPN7,GOSR2,DNM1L,PRKCB

Genes with shortened 3'UTRs are those with significant shortening (FDR = 5%, SAAP analysis) between 4w and 2w samples.

Table S2. Statistics of 3'READS data.

Sample	No. of PASS reads
1w	4,598,547
2w	4,236,999
3w	4,593,749
4w	5,225,934
5w	6,005,976
6w	6,999,620

Figure S1

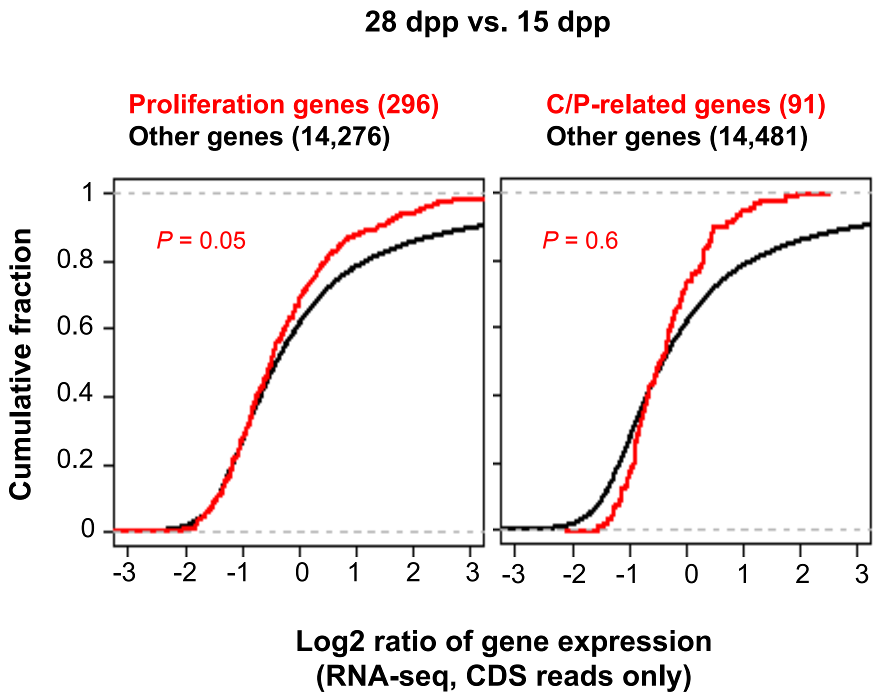

28 dpp vs. 15 dpp
Proliferation genes (296)
Other genes (14,276)
C/P-related genes (91)
Other genes (14,481)
P = 0.05
P = 0.6
Cumulative fraction
1
0.8
0.6
0.4
0.2
0
-3
-2
-1
0
1
2
3
Log2 ratio of gene expression
(RNA-seq, CDS reads only)

Figure S2

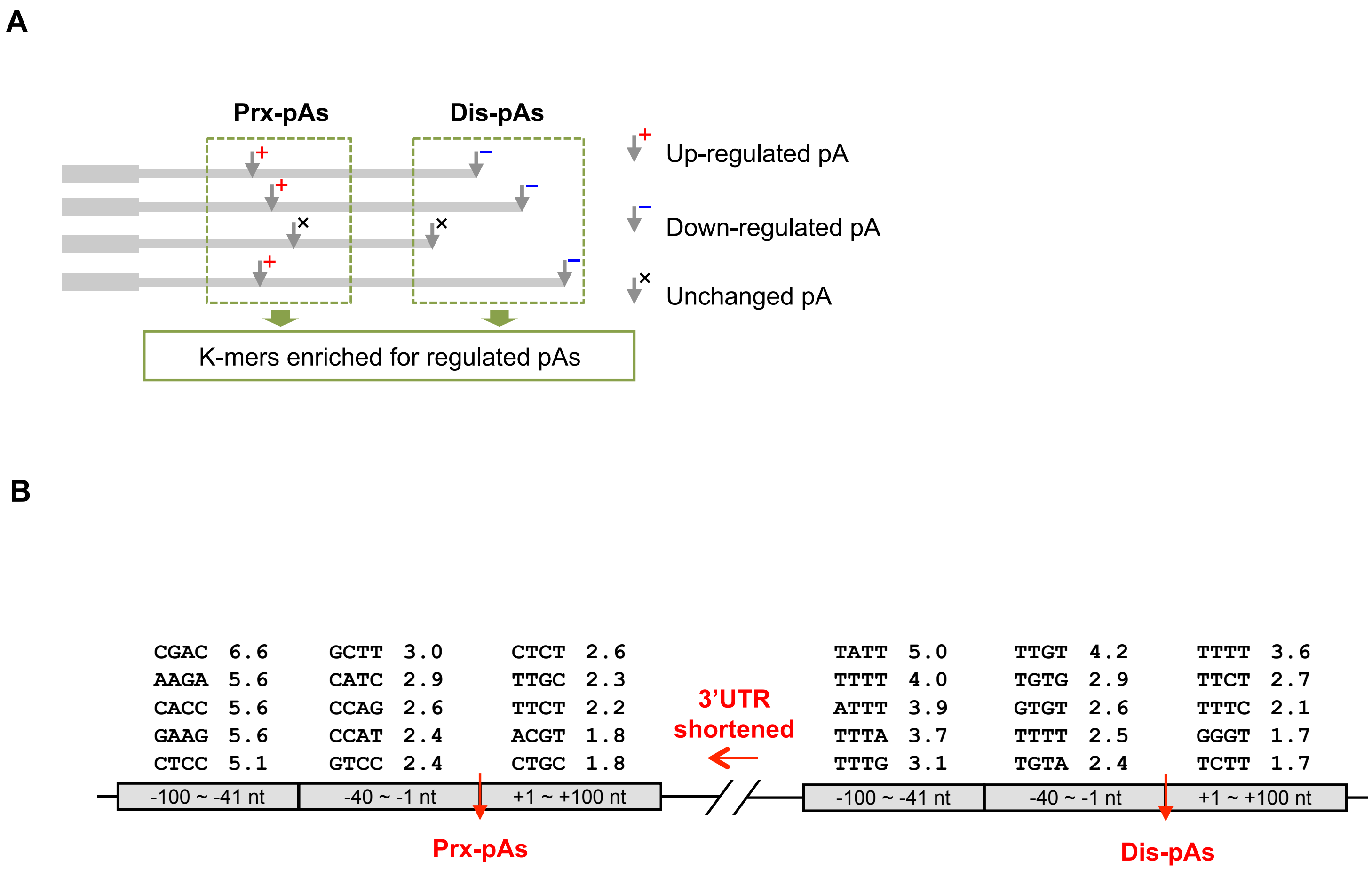
A
Prx-pAs
Dis-pAs
Up-regulated pA
Down-regulated pA
Unchanged pA
K-mers enriched for regulated pAs
B
CGAC 6.6
AAGA 5.6
CACC 5.6
GAAG 5.6
CTCC 5.1
GCTT 3.0
CATC 2.9
CCAG 2.6
CCAT 2.4
GTCC 2.4
CTCT 2.6
TTGC 2.3
TTCT 2.2
ACGT 1.8
CTGC 1.8
3'UTR shortened
TATT 5.0
TTTT 4.0
ATTT 3.9
TTTA 3.7
TTTG 3.1
TTGT 4.2
TGTG 2.9
GTGT 2.6
TTTT 2.5
TGTA 2.4
TTTT 3.6
TTCT 2.7
TTTC 2.1
GGGT 1.7
TCTT 1.7
-100 ~ -41 nt
-40 ~ -1 nt
+1 ~ +100 nt
-100 ~ -41 nt
-40 ~ -1 nt
+1 ~ +100 nt
Prx-pAs
Dis-pAs

Figure S3

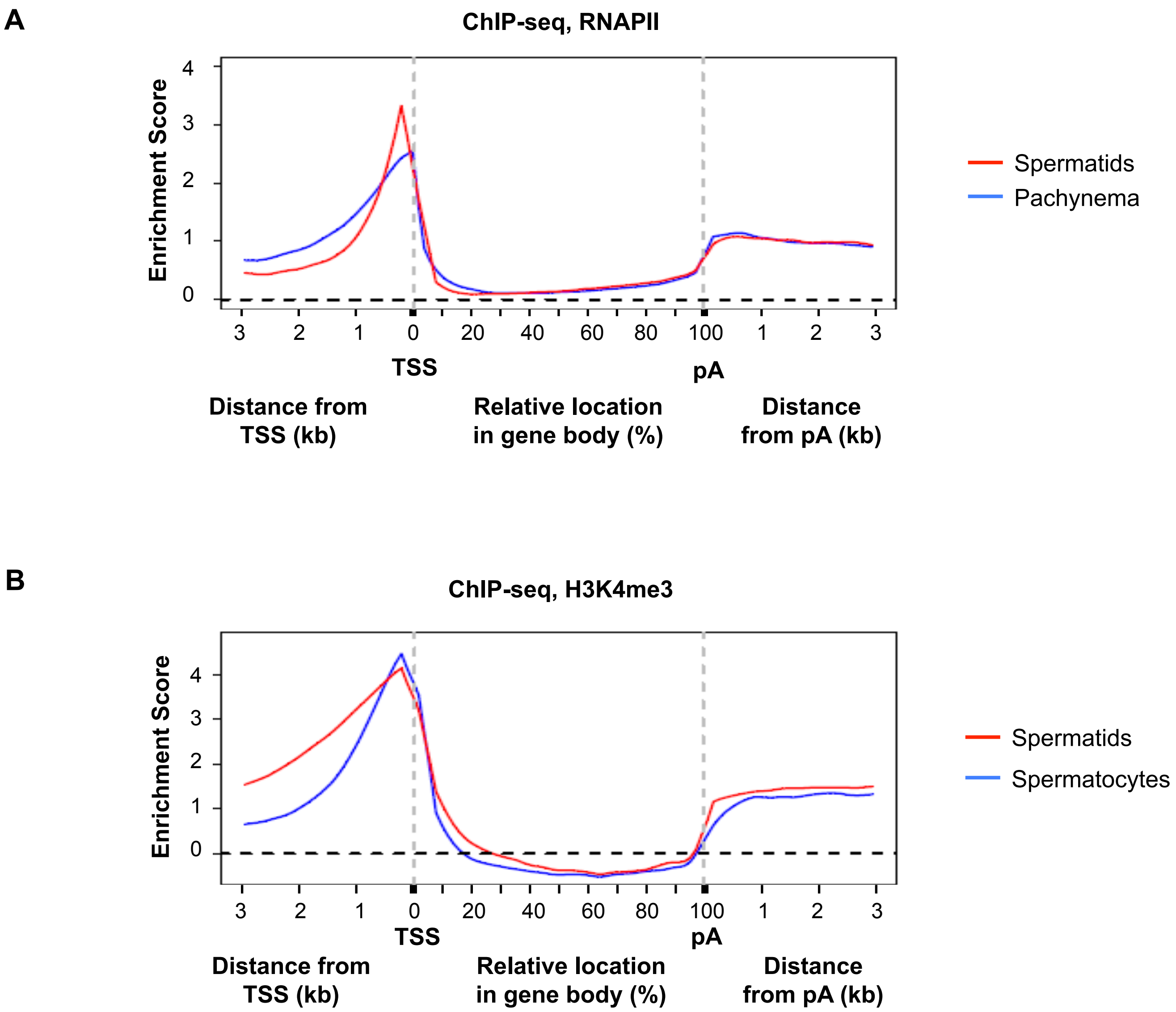
A
ChIP-seq, RNAPII
Enrichment Score
4
3
2
1
0
3
2
1
0
20
40
60
80
100
1
2
3
TSS
pA
Distance from TSS (kb)
Relative location in gene body (%)
Distance from pA (kb)
Spermatids
Pachynema
B
ChIP-seq, H3K4me3
Enrichment Score
4
3
2
1
0
3
2
1
0
20
40
60
80
100
1
2
3
TSS
pA
Distance from TSS (kb)
Relative location in gene body (%)
Distance from pA (kb)
Spermatids
Spermatocytes

Figure S4

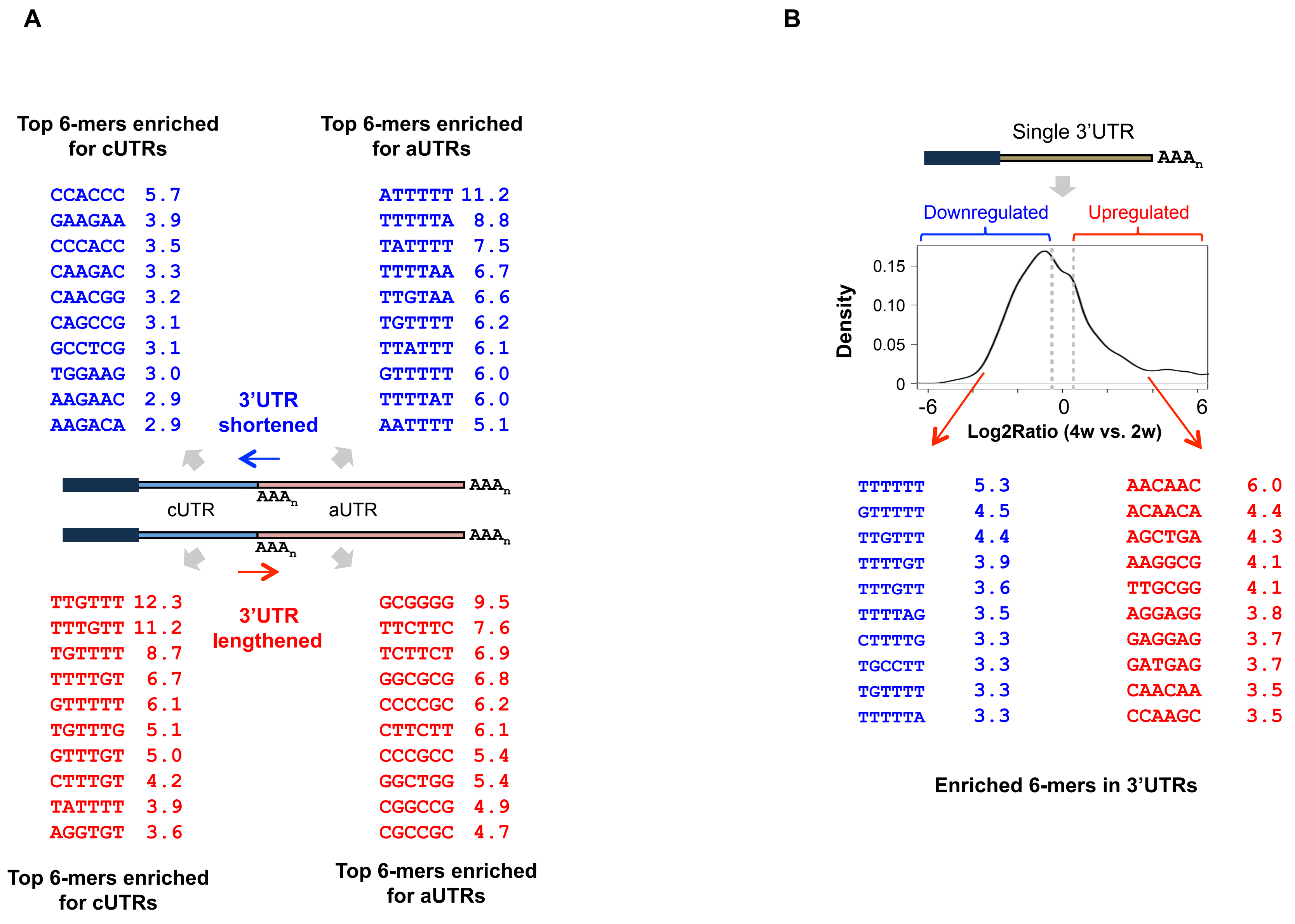
A
Top 6-mers enriched for cUTRs
CCACCC 5.7
GAAGAA 3.9
CCCACC 3.5
CAAGAC 3.3
CAACGG 3.2
CAGCCG 3.1
GCCTCG 3.1
TGGAAG 3.0
AAGAAC 2.9
AAGACA 2.9
Top 6-mers enriched for aUTRs
ATTTTT 11.2
TTTTTA 8.8
TATTTT 7.5
TTTTAA 6.7
TTGTAA 6.6
TGTTTT 6.2
TTATTT 6.1
GTTTTT 6.0
TTTTAT 6.0
AATTTT 5.1
3'UTR shortened
AAA$_n$
cUTR
aUTR
3'UTR lengthened
TTGTTT 12.3
TTTGTT 11.2
TGTTTT 8.7
TTTTGT 6.7
GTTTTT 6.1
TGTTTG 5.1
GTTTGT 5.0
CTTTGT 4.2
TATTTT 3.9
AGGTGT 3.6
GCGGGG 9.5
TTCTTC 7.6
TCTTCT 6.9
GGCGCG 6.8
CCCCGC 6.2
CTTCTT 6.1
CCCGCC 5.4
GGCTGG 5.4
CGGCCG 4.9
CGCCGC 4.7
Top 6-mers enriched for cUTRs
Top 6-mers enriched for aUTRs
B
Single 3'UTR
AAA$_n$
Downregulated
Upregulated
Density
0.15
0.10
0.05
0
-6
0
6
Log2Ratio (4w vs. 2w)
TTTTTT 5.3
GTTTTT 4.5
TTGTTT 4.4
TTTTGT 3.9
TTTGTT 3.6
TTTTAG 3.5
CTTTTG 3.3
TGCCTT 3.3
TGTTTT 3.3
TTTTTA 3.3
AACAAC 6.0
ACAACA 4.4
AGCTGA 4.3
AAGGCG 4.1
TTGCGG 4.1
AGGAGG 3.8
GAGGAG 3.7
GATGAG 3.7
CAACAA 3.5
CCAAGC 3.5
Enriched 6-mers in 3'UTRs

Figure S5

A

Gene expression change, 4w vs. 2w, (3'READS), non-TE sequences only

	4-mers		6-mers	
K-mers enriched for 3'UTRs of upregulated genes			TTGCGG	4.4
			AAGGCG	4.2
			AGCTGA	4.2
			TTCAAG	3.9
			CCAAGC	3.7
	ACCA	7.7	CGGGAT	3.6
	AAGA	6.6	AACAAC	3.5
	CAAG	6.1	GATGAG	3.4
	GAAG	5.8	AACATC	3.3
	TCAA	5.6	GGCAAA	3.2
K-mers enriched for 3'UTRs of downregulated genes	TTTT	10.3	GTTTTT	3.8
	TTGT	7.3	TTTTTT	3.6
	GTTT	7.0	CTTTTG	3.2
	TGGG	7.0	GGTGGG	3.2
	TTTG	5.4	TGCCTT	3.2
			TTGTTT	3.2
			CTTGTT	3.1
			TATTTA	3.0
			TTTTAG	3.0
			TTTTTA	2.9

B

Gene expression change, Miwi -/- vs. Miwi+/-, early round spermatids (RNA-seq)

	4-mers		6-mers	
K-mers enriched for 3'UTRs of upregulated genes			GCAGAG	4.0
			GCTGTC	3.7
			TGAACT	3.4
			GCGGTG	3.1
			CTGGCT	2.8
	CTGG	5.1	GACTGC	2.8
	CCTG	4.3	CAGGGG	2.6
	GCAG	4.3	CCCACC	2.6
	GCCT	3.6	GGCCTG	2.6
	CAGG	3.5	ACTCAC	2.5
K-mers enriched for 3'UTRs of downregulated genes	AAAA	8.6	TGAATA	5.0
	GAAA	5.4	CAAGAA	4.0
	AGAA	4.9	AAGAAA	3.6
	TCAA	4.7	ATGTTC	3.5
	ATCA	4.2	AGAAAA	3.4
			ATGACA	3.3
			AAAAGA	3.2
			GACAAG	3.2
			CAAAAT	3.0
			AAAGAA	2.9

Figure S6

A

upregulated intronic pAs

Intron Location:	Intron size +1	Intron size +2	Intron size M	Intron size -2	Intron size -1	5'SS strength +1	5'SS strength +2	5'SS strength M	5'SS strength -2	5'SS strength -1	3'SS strength +1	3'SS strength +2	3'SS strength M	3'SS strength -2	3'SS strength -1
3w vs. 2w	2.5	1.7	0.4	1.8	1.3	1.6	-0.1	0.3	0.0	-0.1	0.8	0.2	0.7	0.6	0.5
4w vs. 3w	2.7	2.9	1.2	0.8	0.5	0.9	-0.3	1.7	0.8	0.1	0.5	1.1	0.0	0.3	0.8

B

downregulated intronic pAs

Intron Location:	Intron size +1	Intron size +2	Intron size M	Intron size -2	Intron size -1	5'SS strength +1	5'SS strength +2	5'SS strength M	5'SS strength -2	5'SS strength -1	3'SS strength +1	3'SS strength +2	3'SS strength M	3'SS strength -2	3'SS strength -1
3w vs. 2w	-1.2	-1.0	0.9	0.8	0.8	-0.5	-0.4	-1.2	1.4	-0.3	-1.2	0.3	-0.8	-0.8	-0.4

Significance score (vs. background)

Smaller size or Weaker 5'/3'SS

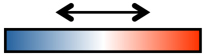

Larger size or Stronger 5'/3'SS

Figure S7

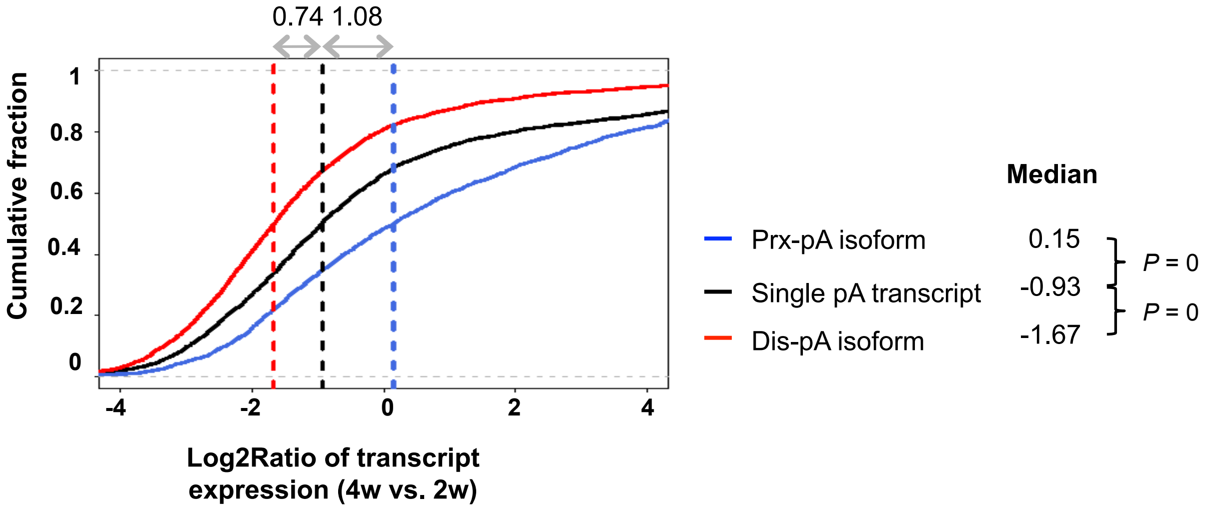

0.74
1.08
Cumulative fraction
1
0.8
0.6
0.4
0.2
0
-4
-2
0
2
4
Log2Ratio of transcript expression (4w vs. 2w)
Median
Prx-pA isoform
0.15
Single pA transcript
-0.93
Dis-pA isoform
-1.67
P = 0
P = 0
